# Burkitt lymphoma of the ovaries mimicking sepsis: a case report and review of the literature

**DOI:** 10.1186/s13256-018-1828-3

**Published:** 2018-10-05

**Authors:** Athanasios Gravos, Konstantinos Sakellaridis, Paraskeui Tselioti, Konstantina Katsifa, Varvara Grammatikopoulou, Aikaterini Nodarou, Κonstantinos Sarantos, Alexandros Tourtoglou, Eirini Tsovolou, Charilaos Tsapas, Athanasios Prekates

**Affiliations:** Intensive Care Unit, Tzaneio General Hospital of Piraeus, Dodonis 26, 13451 Kamatero, Greece

**Keywords:** Burkitt’s lymphoma, Ovaries, Septic shock

## Abstract

**Background:**

It is not unusual for systemic diseases to mimic sepsis and, in any case, the clinician should thoroughly investigate this possibility.

**Case presentation:**

We present the case of a 21-year-old Greek woman who presented to the Intensive Care Unit of our hospital with severe septic shock – multiple organ failure as a result of a suspected gynecological infection of the ovaries. An immediate improvement of her clinical condition in combination with strong clinical suspicion and negative cultures led to the differential diagnosis of diseases other than sepsis. Based on the results of the biopsies that were obtained by research laparotomy, our patient suffered from primary Burkitt ovarian lymphoma. Her clinical condition improved with supportive treatment and chemotherapy. Chemotherapy is the dominant treatment for Burkitt’s lymphoma, while surgery or radiotherapy has no place.

**Conclusions:**

All intensivists should be aware of clinical conditions that mimic sepsis as early diagnosis can lead to appropriate therapy and avoid unnecessary diagnostic tests and antibiotic abuse.

## Background

Primary bilateral non-Hodgkin lymphoma of the ovaries, a subtype of Burkitt’s lymphoma, was first observed by L. R. Weekes in a 15-year-old Guatemalan girl in 1986 [1]. Primary ovarian lymphomas account for 0.5% of all non-Hodgkin lymphomas and 1% of all ovarian neoplasms [2]. Almost all cases of Burkitt’s lymphoma of the ovaries involve both ovaries. The use of surgery in the treatment of Burkitt’s lymphoma is debatable with present day chemotherapy regimens [3]. The prognosis is excellent and appropriate preoperative diagnosis can avoid extensive and unnecessary surgery in these patients.

The clinical presentation is extremely heterogeneous [4]. The mild forms appear with progressive swelling of the glands, usually in many areas from the beginning, swelling of the spleen, without fever, and they evolve slowly. However, in many cases they turn into acute leukemia, which is difficult to treat and is often unsuccessfully treated. Several forms are initially aggressive with rapid and large swollen lymph nodes, often resulting in pressure on other organs (ureter occlusion, brain and spinal cord pressure, and so on), pleural and pericardial infiltration, testicular swelling/infiltration, bone destruction, gonads and endocrine glands infiltration, but also B-symptoms (fever, weight loss, sweating).

Chemotherapy is the dominant treatment for Burkitt’s lymphoma [5]. Intravenously administered antibiotics for neutropenic fever and growth factors, that is, granulocyte-macrophage colony-stimulating factor (GM-CSF) or granulocyte-colony-stimulating factor (G-CSF), are administered to reduce the duration of neutropenia. Surgery or radiotherapy has no place in the treatment of Burkitt’s lymphoma.

## Case presentation

### Patient information

We present the case of a 21-year-old Greek woman who presented to the Emergencies Department of our hospital with breast pain, abdominal distension, and weakness of approximately 1 week’s duration. Her individual, gynecological, and family history were unremarkable.

### Clinical findings

She had a high breathing rate (~ 22 breaths/minute), tachycardia (~ 110 beats/minute), hypotension with mean arterial pressure (MAP) of 55 mmHg, lethargy, swollen and painful breasts, abdominal dilatation with diffuse sensitivity to palpation and dullness on percussion, and low grade fever (~ 37.5 °C).

### Diagnostic assessment

**Fig. 1:**
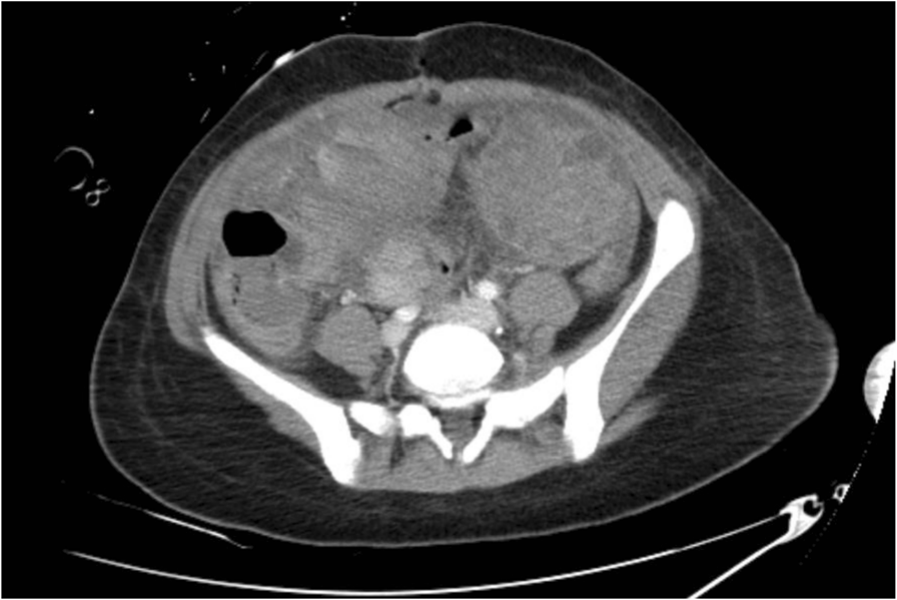
Image of the ovaries of the patient from the computed tomography scan

She was directly subjected to ultrasound (U/S) of her upper and lower abdomen that showed enlarged ovaries as well as a large amount of free ascitic fluid. Complete laboratory testing and blood gases were obtained and an urgent computed tomography (CT) scan of her upper and lower abdomen was performed, confirming the findings of the U/S: enlarged and inflammatory ovaries, pleural effusions, and large amount of free ascitic fluid (Fig. 1). Laboratory tests showed neutrophilic leukocytosis with white blood cells (WBC) 30,000/μL, polymorphonucleocytes (PMN) 95%, and thrombocytopenia with platelets (PLT) 90,000/μL with signs of disseminated intravascular coagulation (DIC), increased urea (U) and creatinine (Cr) levels, increased bilirubin (Bil), increased serum glutamic oxaloacetic transaminase (SGOT) and serum glutamic pyruvic transaminase (SGPT), and severe lactic and metabolic acidosis. Control for viral and human immunodeficincy virus (HIV) infection was negative. With these data and due to further deterioration of our patient’s clinical condition, it was decided to conduct a research laparotomy.

Intraoperatively there were found enlarged and inflamed ovaries, a large amount of ascitic fluid, and an inflamed appendix, which was removed and sent for biopsy. The peritoneum was clear and free of any visible implants. A biopsy was also obtained from both her ovaries and cytology and ascitic fluid cultures were sent, a suprapubic fluid drainage tube was placed and due to the reproductive age of our patient it was decided not to remove her ovaries. After surgery, she was transported to the Intensive Care Unit (ICU), intubated, and mechanically ventilated; she was hemodynamically unstable, presenting hypoxemia with partial pressure of oxygen in arterial blood/fraction of inspired oxygen (PaO_2_/FiO_2_) of 150 and severe metabolic and lactic acidosis.

The differential diagnosis included ovarian hyperstimulation syndrome (OHSS) and other ovarian tumors. OHSS is a systemic disorder attributed to the release of vasoactive agents released from the ovaries after overstimulation [6]. The pathophysiology of OHSS is characterized by increased capillary permeability leading to large fluid extravasation, accumulation in the third space, and intravascular dehydration [7]. The serious manifestations of the syndrome include thrombosis, renal and hepatic insufficiency, and acute respiratory distress syndrome (ARDS), which cause severe morbidity [8]. Mortality from the syndrome is fortunately rare, with only sporadic references in the literature [8]. Women should be aware that mild forms of OHSS are common and complicate 33% of *in vitro* fertilization (IVF) cycles, while moderate and severe forms occur in 3–8% of cases of OHSS [6]. The majority of serious OHSS cases occur after IVF cycles, but the syndrome may also occur after any form of ovulation induction, such as clomiphene and gonadotropins [6]. The incidence of the syndrome, particularly the complex form, is higher in young women, women with polycystic ovaries, and in gestational cycles. Laboratory tests may show high hematocrit levels (> 55%), hypoproteinemia, and leukocytosis [8]. The treatment of OHSS is initially supportive until the situation resolves [9].

### Therapeutic interventions

She was initially treated as severe septic shock; blood cultures were obtained and broad-spectrum antibiotic treatment was administered. Due to acute renal failure, she was placed in continuous venous-venous hemofiltration (CVVHDF).

While she was in our ICU she showed progressive clinical, gasometric, and hemodynamic improvement, draining ~ 2000 ml of ascitic fluid/day; on the third day of admission an attempt was made to wean her from the ventilator, pending the results of the cultures and ovarian and appendix biopsies. She was febrile (~ 38.4 °C), hemodynamically stable with normal hourly diuresis, and improved laboratory testing, therefore CVVHDF was removed. Severe leukopenia (WBC 2000/μL) was evident, for which she received subcutaneous granulocyte growth factor. On the fourth day of admission, the results of blood and ascites fluid cultures were negative and biopsy results showed high-grade Burkitt lymphoma of the ovaries and the appendix. With these data our patient was transported to a specialized oncology center for immediate onset of chemotherapy and further treatment.

### Follow-up and outcomes

She was gradually weaned from mechanical ventilation and was successfully extubated on the 12th day of her hospitalization. On the sixth day she received a combined chemotherapy regimen intravenously. On the 15th day she left the ICU and on the 28th day she was discharged from hospital, presenting improved clinical and laboratory condition, waiting for further cycles of chemotherapy.

## Discussion

We present the case of a patient with clinical presentation of severe septic shock and multiple organ failure syndrome due to a suspected internal inflammatory disease. Initially, while waiting for blood and biopsy results, she was treated for septic shock, but due to the immediate improvement in her clinical condition and hemodynamic instability, the differential diagnosis turned to other diseases, such as OHSS and low differentiation lymphomas. It is not uncommon that ICUs deal with diseases that mimic sepsis and are initially treated as sepsis, pending the results of blood cultures and biopsies. Detailed history and strong clinical suspicion require further testing in cases where sepsis evolves and improves rapidly, and blood and tissue cultures are negative, despite non-surgical removal of the source of the suspected infection.

In our case, our patient suffered from undiagnosed Burkitt lymphoma of the ovaries and the appendix. Burkitt’s lymphoma accounts for 0.8% of all B-lymphomas [10]. It occurs predominantly in children and young people. There are three forms [10]. (a) The endemic form, which is present in Africa, particularly in areas with malaria, is directly related to Epstein–Barr virus (EBV) infection and is found in the bones of the face and out of lymph nodes. Pathogenetically, it is attributed to the overexpression of the oncogene *c-MYC* [11] (a strong transcription factor that promotes proliferation) when the end portion of the chromosome 8 translocates and comes into immediate proximity with the immunoglobulin G heavy chain promoters that are based on chromosome 14. (b) The sporadic form, which is mainly localized in the ileocecal region and in the gonads (our case), is associated with a lower percentage of EBV infection and responds well to the appropriate chemotherapy. (c) There is a form associated with immune deficiency, which occurs mainly in patients with HIV infection, regardless of the number of CD4 cells.

On histological examination, the cells are small, round-core, showing a diffuse growth model and in the presence of several macrophages/dendritic cells they give a starry sky image [10]. Patients with > 25% involvement of the bone marrow are considered to have Burkitt leukemia [11].

Laboratory testing often shows anemia due to marrow infiltration or autoimmune hemolytic anemia, leukopenia, and thrombocytopenia due to marrow infiltration, spleen entrapment, and autoimmune destruction mechanisms but also as a consequence of chemotherapy. Biochemical tests must include lactate dehydrogenase (LDH) and β2-microglobulin, which are also reliable diagnostic and response criteria markers, C-reactive protein (CRP), uric acid, and other tests related to organ damage from the lymphoma. The laboratory tests are supplemented by the following specific tests: (a) immunochemistry tests, (b) cytogenetic studies, and (c) molecular techniques [4].

The diagnostic workup with appropriate imaging techniques and laboratory tests should lead without delay to the biopsy of one of the affected glands. Fine-needle biopsy can be used for swelling of the abdominal or thoracic lymph nodes, while an organ biopsy can be obtained (as in our case). Necessary supplementation is the biopsy of the bone marrow, which can highlight or exclude the infiltration of the marrow from the lymphoma. In our case, bone marrow was not involved. The marrow extract can be studied by polymerase chain reaction (PCR). Bone marrow infiltration implies a worse prognosis.

The following stages are distinguished (Ann Arbor Staging) [12]:

- Stage I. Inflammation of lymph nodes in a region or an extranodal area.
- Stage II. Single tumor (extranodal) with regional node involvement. Lymphoma involving nodal areas on the same side of the diaphragm.
- Stage III. Lymphoma involving sites on opposite sides of the diaphragm. All primary intrathoracic tumors. All paraspinal or epidural tumors. Extensive intra-abdominal disease (our case).
- Stage IV. Any of the above, with central nervous system (CNS) or bone marrow involvement (< 25%) at presentation.
  A: No general symptoms
  B: With general symptoms (weight loss, fever, sweats).

The gathering of the above information allows the classification of patients in stages and determines prognosis, leading to the decision to initiate treatment, especially in slowly evolving lymphomas.

In general, there are three approaches to chemotherapy for Burkitt’s lymphoma [13]:

1. Intensive short-term chemotherapy.
2. Long-term chemotherapy similar to treatment of acute lymphoblastic leukemia.
3. Combinations followed by autologous stem cell transplantation.

Most existing regimens add rituximab to chemotherapy regimens [14]. Other medicines that can be used in patients with Burkitt’s lymphoma include: glucocorticoids (for example, prednisone), urate oxidase enzymes (for example, rasburicase), prophylactic allopurinol, and aggressive hydration with urine alkalinization (to reduce the risk of tumor lysis syndrome and uric acid nephropathy).

Approximately 90% of pediatric patients and 50–60% of adult patients with Burkitt’s lymphoma receiving chemotherapy-intensive regimens have long-term disease-free survival [15]. The prognosis of Burkitt’s lymphoma in children is correlated with the disease volume at the time of diagnosis.

Patients with limited disease (Stages I and II) have an excellent prognosis with a survival rate greater than 90% [13]. Patients with more extensive disease (Stages III and IV), particularly with bone marrow and CNS involvement, have a worse prognosis, but long-term survival rates of 50–90% can be achieved with more aggressive chemotherapy regimens. Patients with recurrence have a long-term survival rate of 20–50% [15]. Adding rituximab may further increase the response rate [14]. Adults with Burkitt’s lymphoma, especially those with an advanced stage, have a worse prognosis [16].

## Conclusions

We present the case of a patient admitted to our ICU presenting with severe septic shock – multiple organ failure as a result of suspected gynecological infection. Direct patient improvement coupled with negative blood and tissue cultures and strong clinical suspicion led our diagnostic thinking to other diseases. An ovarian biopsy taken intraoperatively revealed Burkitt lymphoma of the ovaries. It is not uncommon for systemic diseases to mimic sepsis and, in any case, clinicians should thoroughly investigate and exclude this possibility.
